# Therapeutic Effects of Myriocin in Experimental Alcohol-Related Neurobehavioral Dysfunction and Frontal Lobe White Matter Biochemical Pathology

**DOI:** 10.4236/jbbs.2022.122003

**Published:** 2022-02-10

**Authors:** Camilla Homans, Emine B. Yalcin, Ming Tong, Gina Gallucci, David Bautista, Natalia Moriel, Suzanne de la Monte

**Affiliations:** 1 Biotechnology Graduate Program, Brown University, Providence, RI, USA; 2 Department of Pathology and Laboratory Medicine, Warren Alpert Medical School of Brown University, Providence, RI, USA; 3 Liver Research Center, Department of Medicine, Rhode Island Hospital, Providence, RI, USA; 4 Warren Alpert Medical School of Brown University, Providence, RI, USA; 5 Brown University, Providence, RI, USA; 6 Department of Pathology and Laboratory Medicine, Rhode Island Hospital, Women and Infants Hospital of Rhode Island, Providence VA Medical Center, Providence, RI, USA

**Keywords:** Adolescence, Alcohol, Behavior, Binge Drinking, Brain Atrophy, Myriocin, Neurodegeneration, Rat, Sulfatide, White Matter

## Abstract

**Background & Objective:**

Chronic excessive alcohol consumption causes white matter degeneration with myelin loss and impaired neuronal conductivity. Subsequent rarefaction of myelin accounts for the sustained deficits in cognition, learning, and memory. Correspondingly, chronic heavy or repeated binge alcohol exposures in humans and experimental models alter myelin lipid composition leading to build-up of ceramides which can be neurotoxic and broadly inhibitory to brain functions.

**Methods:**

This study examined the effects of chronic + binge alcohol exposures (8 weeks) and intervention with myriocin, a ceramide inhibitor, on neurobehavioral functions (Open Field, Novel Object Recognition, and Morris Water Maze tests) and frontal lobe white matter myelin lipid biochemical pathology in an adult Long-Evans rat model.

**Results:**

The ethanol-exposed group had significant deficits in executive functions with increased indices of anxiety and impairments in spatial learning acquisition. Myriocin partially remediated these effects of ethanol while not impacting behavior in the control group. Ethanol-fed rats had significantly smaller brains with broadly reduced expression of sulfatides and reduced expression of two of the three sphingomyelins detected in frontal white matter. Myriocin partially resolved these effects corresponding with improvements in neurobehavioral function.

**Conclusion:**

Therapeutic strategies that support cerebral white matter myelin expression of sulfatide and sphingomyelin may help remediate cognitive-behavioral dysfunction following chronic heavy alcohol consumption in humans.

## 1. Introduction

Chronic heavy and repeated binge alcohol exposures adversely alter brain structure and metabolism across the lifespan [1]. Long-term consequences include cognitive-motor and visual-spatial deficits and impairments in executive function [2], which can progress to dementia [3] [4]. Major brain targets of alcohol’s neurotoxic and neurodegenerative effects include the cerebral cortex (anterior frontal and temporal lobes), hippocampus, white matter, including frontal lobe and corpus callosum, and the cerebellum. Macroscopically, these effects are manifested by atrophy with ventriculomegaly. Although alcohol-related brain degeneration is consistently associated with disproportionate white matter damage [1] [5] [6] [7] [8] [9], studies have remained limited due to technical difficulties with the analysis of myelin, which has a very high dry mass of lipid (70%) compared with protein (30%). However, it is the unusually abundant lipid composition that enables myelin to provide the insulation needed for efficient neurotransmission.

White matter lipids include mainly phospholipids, cholesterol, and sphingolipids. Although chronic alcohol exposures alter most lipid subtypes [10] [11], sphingolipids are of particular interest because of their functional roles in cognitive and motor activities, lipid rafts, and signal transduction [12] [13] [14] [15] [16]. Alcohol has been shown to reduce expression of sphingomyelin and sulfatide while increasing expression of ceramides in white matter [10] [17] [18]. Low levels of ceramide are needed for normal brain functions including metabolism, but high levels can be neurotoxic, inhibit signaling through cell survival and metabolic pathways, increase oxidative stress and mitochondrial dysfunction, and promote neuroinflammation [19]. On the other hand, reduced expression of sulfatide or sphingomyelin in various disease states, including after chronic alcohol exposures, has been linked to demyelination and cell death with impairments in cognition, learning, memory, and performance [19] [20]. Pathophysiological states that increase sphingomyelin degradation or sulfatide catabolism via arylsulfatase A and galactosylceramidase promote ceramide accumulation. Correspondingly, experimental administration of short-chain ceramides impairs spatial learning and memory and increases molecular and biochemical indices of neurodegeneration in the brain [21]. These findings suggest that alcohol-related white matter degeneration could potentially be halted or reversed by agents that prevent accumulation of ceramides in the brain.

Myriocin is a fungus-derived natural product that has been incorporated into traditional Chinese medicine to achieve eternal youth. Myriocin was approved by the United States Food and Drug Administration (FDA) under the names, “ISP-1” and “FTY720” and is now an oral drug used for inflammatory demyelinating diseases including multiple sclerosis [22]. One of its main positive effects is to inhibit T cell-mediated inflammation [23]. However, at high doses, it can inhibit protein kinase C activity and suppress growth of tumor or normal cells [23] [24] [25]. Biochemically, myriocin disrupts ceramide synthesis by blocking the essential enzyme serine palmitoyltransferase. In previous human and experimental models of steatohepatitis caused by chronic alcohol exposure or obesity with metabolic syndrome, myriocin treatment resolved the hepatic architectural and molecular pathologies [26]. The present research project was designed to determine if similar effects of myriocin occurred in frontal cerebral white matter of an established model of alcohol-related white matter degeneration and whether the treatments improved cognitive behavioral functions and normalized white matter sphingolipid profiles.

## 2. Methods

### 1) General:

The Institutional Animal Care and Use Committee (IACUC) at the Lifespan/Rhode Island Hospital approved the use of rats for this experiment. Throughout the experiment, rats were housed under standardized humane conditions and kept on a 12-hour light/dark cycle with free access to food. All experiments were performed in accordance with our IACUC approved protocols and conformed with guidelines established by the National Institutes of Health.

### 2) Experimental Model:

Long Evans male and female 4 weeks old rats (Harlan Sprague Dawley, Inc., Indianapolis, IN, USA) were subdivided to generate a 4-way model to examine the effects of chronic + binge alcohol feeding [11] and myriocin treatment [26] on cognitive-behavioral functions and white matter biochemical pathology. We used a chronic + binge instead of a standard chronic model of ethanol feeding because previous studies demonstrated it to be more effective for producing alcohol-mediated liver injury and simulating human disease [27] [28]. Males and females, selected at random, were included in all groups, each of which contained 6 rats. Two sub-groups were maintained for 8 weeks on 24% ethanol-containing (caloric content) Lieber-DeCarli liquid diets (BioServ, Frenchtown, NJ USA), and during the last 3 weeks, they were gavaged (binged) with 2 g/kg ethanol in a liquid diet (2.5 mL total volume) late in the afternoon on Tuesdays, Thursdays, and Saturdays. The two control groups were simultaneously maintained on isocaloric liquid diets containing 0% ethanol, and during Weeks 5 - 8, they were gavaged with saline in liquid diet (2.5 mL total volume).

### 3) Therapeutic Intervention:

**Figure 1.**
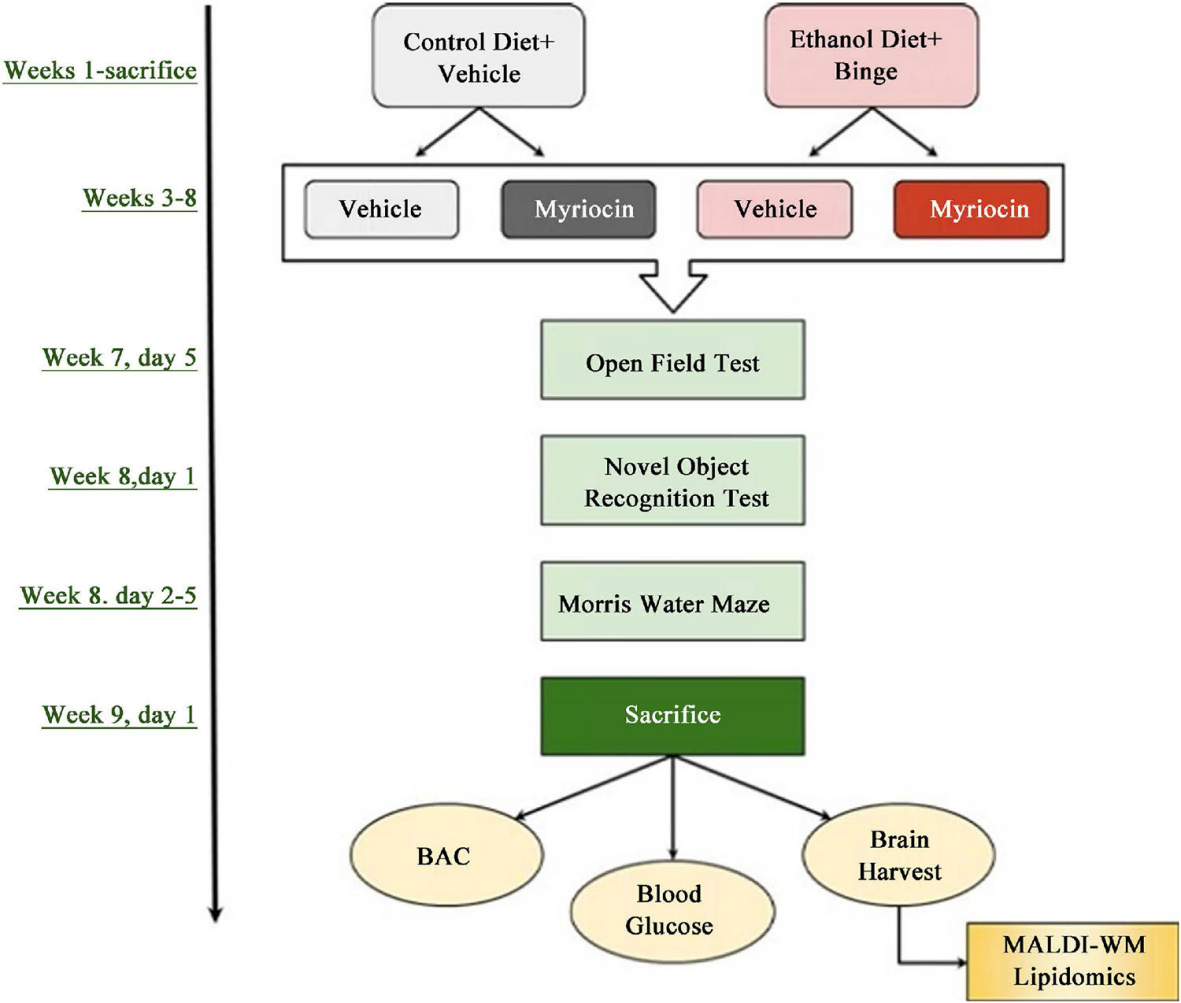
Experimental Model: A 4-way 8-week model of chronic (24% ethanol, caloric content) + binge (2 g/Kg, gavage) ethanol or isocaloric control (0% ethanol + saline gavage) liquid diet feeding with (0.3 mg/kg, i.p.) or without (saline, i.p.) myriocin treatment was generated in 4-week old Long Evans male and female rats as depicted in the diagram. Binge ethanol or vehicle administrations were performed on Tuesday, Thursday, and Saturday afternoons of Weeks 6 - 8, and myriocin (or vehicle) treatments were administered on Monday, Wednesday, and Friday of Weeks 3 through 8. Neurobehavioral tests including Open Field (OF), Novel Object Recognition (NOR), and Morris Water Maze (MWM) tests were performed during Weeks 7 and 8, in the mornings, prior to ethanol/saline gavages and i.p. myriocin/saline injections. Upon sacrifice, blood samples were obtained and brains were harvested.

Previous studies showed that 2 g/kg ethanol binge dosing raised blood alcohol to levels detected in humans who binge drink (http://www.clinlabnavigator.com/alcohol-ethanol-ethyl-alcohol.html), yet it safely and effectively causes significant alcohol-related organ pathology [29]. For therapeutic interventions, after 3 weeks on the liquid diets, one ethanol and one control group were given intraperitoneal i.p.) injections of myriocin (0.3 mg/kg in 50 *μ*l) (Sigma-Aldrich Co, St. Louis, MA) while the other two were injected with saline (vehicle) on Mondays, Wednesdays, and Fridays for the duration of the study. See flow diagram in Figure 1.

The alternate day schedule for gavage binging and i.p. myriocin was designed to minimize potential interactions between the acute alcohol administration and drug. Alcohol can alter the pharmacokinetics of various drugs including absorption and metabolism, and alcohol’s pharmacokinetics can be altered by drugs [30] [31] [32]. In humans, the elimination half-life of the FTY720 synthetic structural analogue of myriocin ranges between 89 and 157 hours, irrespective of dose [33] [34].

#### a) Model Analysis:

Rats were monitored daily to ensure adequate food intake, and weekly for body weight. During the mornings of the final week of the experiment, all rats were subjected to three sets of neurobehavioral testing. On Monday, the rats were evaluated with the Open Field and Novel Object Recognition tests, and from Tuesday through Friday, they were evaluated by Morris Water Maze testing (see details below). All rats were fully awake and alert and exhibited no signs of intoxication at testing. Ample breaks (at least 10 minutes) were allowed between tests. At the end of the experiment (at sacrifice), morning blood alcohol levels (cardiac) were measured using an Analox GM7 Analyzer (Analox Instruments, Lunenburg, MA) and blood glucose was measured with a One-Touch II glucometer (Lifescan Inc, Milpitas, CA) in all rats. The rats were then sacrificed by cardiac puncture and exsanguination under deep isoflurane anesthesia. The brains were harvested immediately, and a standardized 3-mm thick coronal slice made from a cut just anterior to the temporal poles and a second cut just posterior to the infundibulum, was stored frozen at −80°C for later studies.

#### b) Open Field Test:

Open Field (OF) testing was used to assess exploratory behavior/anxiety and locomotor activities [35] [36] [37]. This was accomplished using a standard size acrylic box equipped with an overhead video camera. The rats were individually placed in the empty field facing the wall and allowed to explore for 5 minutes. Anxiety-like behavior was reflected by the latencies in arriving at the center, time spent in the center, and number of entries into the center. The results were analyzed with Ethovision 13.0 software. Between trials/tests, the OF apparatus was cleaned with 70% ethanol.

#### c) Novel Object Recognition test (NOR):

NOR testing to assess recognition learning and memory, was performed in the same apparatus used for OF testing [38]. After an initial 5-minute period of acclimation, the familiarization phase was initiated by placing two identical objects side by side onto the maze floor and permitting the rats 10 minutes to explore and get familiarized with the objects. Then, 2 hours later, one of the original objects was replaced with a novel object and the rats were permitted 10 minutes of exploration. The number and percentage of time spent investigating the novel object relative to the familiar object, *i.e.* preference for the novel object, was recorded as an index of memory. Orientation-based preference was minimized by placing the rats into the box facing the wall opposite the objects.

#### d) Morris Water Maze:

MWM testing was used to assess spatial learning and memory [39]. In brief, rats were subjected to 3 daily trials in which the latencies (seconds) required to locate and land on the platform were recorded. On Day 1, the platform was visible, but on Days 2 - 4, the platform was submerged. On Days 3 and 4, the maze entry quadrants were randomized for each trial. The rats were allowed 120 seconds to locate the platform, after which they were guided. Recordings of the trials were analyzed using the Ethovision 13.0 software. Area-under-curve calculations were made for the 3 daily trials. Data were analyzed by ANOVA with post hoc Tukey and linear trend tests.

#### e) Matrix-assisted laser desorption/ionization imaging mass spectrometry (MALDI-IMS):

Detailed methods have been reported elsewhere [11] [17] [40]. In brief, fresh frozen 1.5-mm diameter cores of anterior frontal white matter were excised with a Histoarray tool and embedded in a modified optimum cutting temperature (OCT) medium. Cryosections, 8 μm thick, were thaw-mounted onto indium tin oxide (ITO)-coated slides (Delta Technologies, Loveland, CO), and sublimed with 5-dehydroxybenzoic acid (DHB; Sigma-Aldrich Co, St. Louis, MO) as matrix [17] [40] [41]. External mass calibration was achieved by depositing 1*μ*l of a standard peptide mixture (Peptide Calibration Standard II, Bruker Daltonics) with matrix onto the slide after sublimation. The mixture’s seven calibration points had masses ranging between 377 and 2463 Da, allowing for mass accuracy for sphingolipids. The sections were imaged in the negative ion mode using a reflectron geometry MALDI-time-of-flight (TOF)/TOF mass spectrometer (Ultraflextreme, Bruker Daltonics, Bremen, Germany), and analyzed by focusing a Smartbeam II Nd:YAG laser onto ~75 μm^2^ areas of tissue [17] [40] [42]. Negative ion mode imaging is optimum for detecting sulfatides [43] [44].

#### f) Lipid Analysis:

Sphingolipids were identified by generating production spectra with tandem mass spectrometry (LIFT-TOF/TOF) and fragment ion searches in LIPID MAPS (https://www.lipidmaps.org/). Lipid ion assignments/identifications were made by comparing the precursor and product ion m/z values with catalogued data in LIPID MAPS and confirmed by TOF as previously described [17] [40]. Alternatively, lipid ion assignments were based on published reports [45]-[50].

#### g) Data Analysis.

MALDI data were processed and visualized with Flex-Imaging software v4.0 (Bruker Daltonics, Billerica, MA). Results were normalized to total ion count and analyzed using ClinProTools v3.0 (Bruker Daltonics, Billerica, MA). M/z values for each sample were aligned together in Excel based on their experimental groups. Lipids were identified by their m/z values using literature and the LIPID MAPS database. The m/z values with multiple identifications were further analyzed by MS/MS. Heatmaps provided focused inter-group comparisons. For the heatmap, lipid profiles based on average intensities of expressed lipid ions were compared with respect to ethanol exposure and myriocin treatment in GeneCluster 3.0 [51]. Hierarchical clustering was applied, and the dendrogram was displayed using Java TreeView [51]. Inter-group differences were compared by Two-way ANOVA, and the post hoc Tukey repeated measures test. Principal component analysis (PCA) was used to generate 2-D and 3-D plots to compare the control with experimental groups with respect to their overall patterns of lipid ion expression. Data bar plots generated with Microsoft Excel 2016 Conditional Formatting) (Microsoft Corporation, Redmond, WA, USA) were used to display Inter-group comparisons and the differences in mean lipid expression were analyzed using T-tests with a 1% false discovery rate (GraphPad Prism 8.2, La Jolla, CA, USA).

## 3. Results

### 1) Effects of Ethanol and Myriocin Treatments:

**Figure 2.**
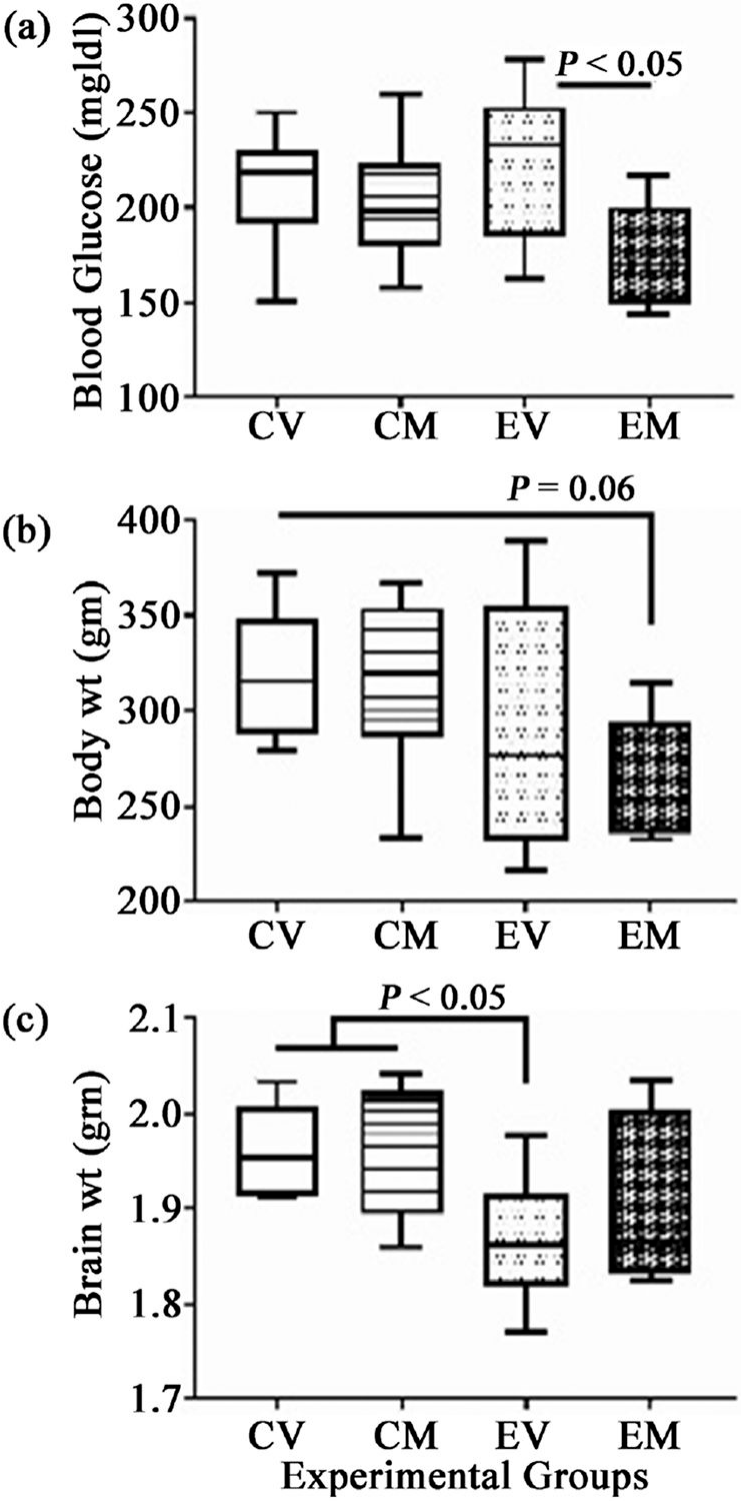
Effects of ethanol and myriocin treatments on (a) blood glucose; (b) body weight; (c) brain weight: A 4-way 8-week experimental model was generated in adult Long Evans male and female rats (n = 8/group) as diagramed in Figure 1. The treatment groups were as follows: CV= control diet + vehicle; CM= control diet + myriocin; EV = ethanol diet + vehicle; EM = ethanol diet + myriocin. All measurements were obtained at sacrifice. The box plots depict the mean (horizontal bar), 95% confidence limits (upper and lower boundaries of the box), and range (stems) corresponding to each parameter measured. Data were analyzed by one-way ANOVA with post hoc Tukey tests. Significant P-values and trends (italicized) are shown in the panels.

Blood alcohol concentrations were similarly elevated in both ethanol groups (±myriocin) (53.4 - 74.65) relative to controls (7.1 - 9.3). Mean blood glucose was highest in the Ethanol + Vehicle (EV), and significantly reduced in the Ethanol + Myriocin (EM) group relative to the other three groups (P < 0.05). The mean blood glucose levels in the control groups were similar and intermediate between those measured in EV and EM (Figure 2). Despite adequate food intake, the mean body weights of ethanol-fed rats were below control. EM’s mean body weight was significantly lower than the other three groups’. Mean brain weight was significantly lower in the EV (1.87 ± 0.06) relative to Control + Vehicle (CV) (1.96 ± 0.04; p < 0.05) and Control + Myriocin (CM) (1.98 ± 0.07; p < 0.05), whereas the mean brain weight for EM (1.90 ± 0.08) did not significantly differ from either group (Figure 2). However, further analysis of sex effects demonstrated that only EV females had a significantly reduced mean brain weight (1.84 ± 0.05) relative to CV females (1.96 ± 0.05; p < 0.05) and CM males (1.98 ± 0.08; p < 0.05). Most important was that myriocin normalized mean brain weight in ethanol-exposed rats but had no significant effect on control brain weight.

### 2) Open Field test (OF):

**Figure 3.**
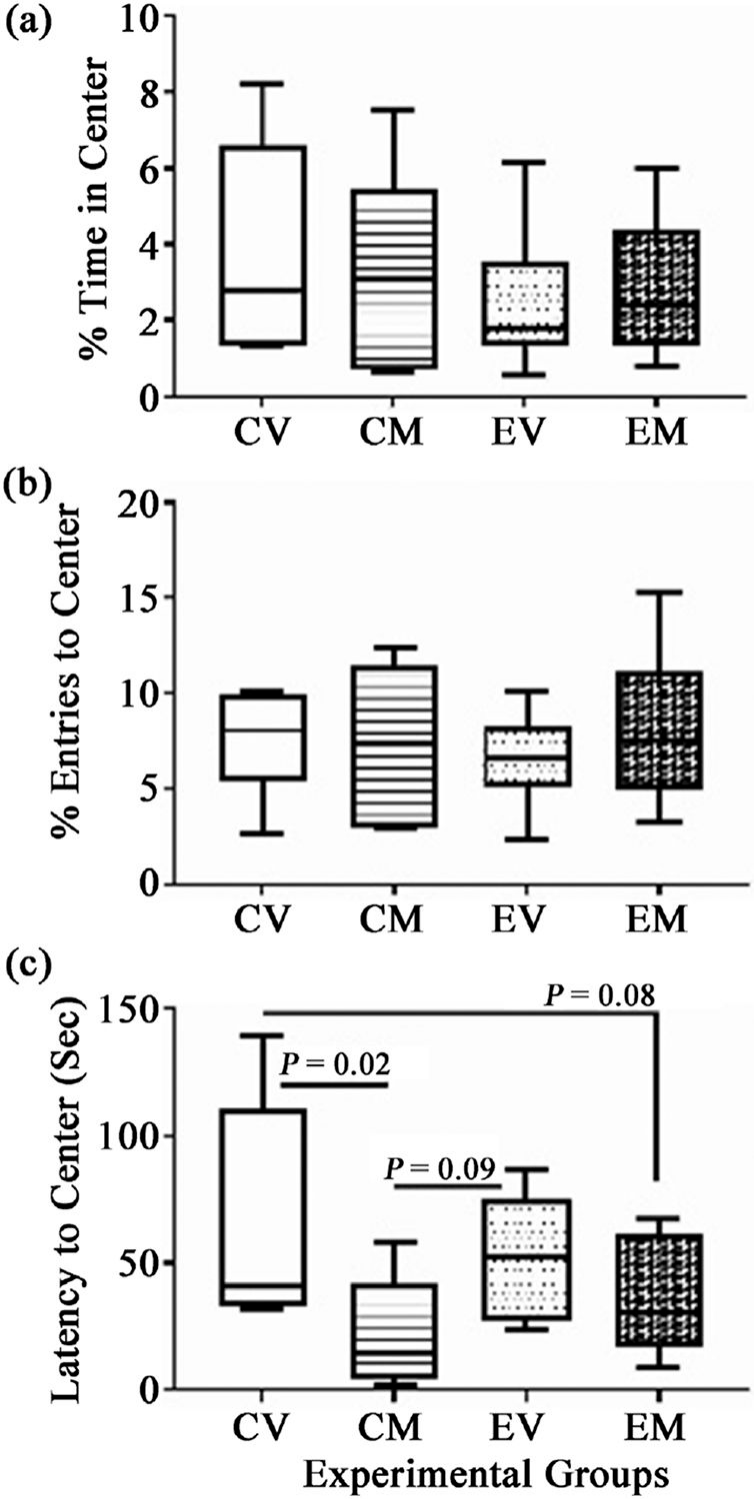
Open Field (OF) Testing: The 4-way, 8-week model generated in male and female Long Evans rats (see Figure 1) was sub-grouped as follows: CV = control diet + vehicle; CM = control diet + myriocin; EV = ethanol diet + vehicle; EM = ethanol diet + myriocin. Performance on the OF test was assessed on Monday of Experimental Week 8 and data were electronically recorded and analyzed (Ethovision 13.0 software) with respect to the (a) percentage of time spent in the center; (b) the percentage of entries into the center; (c) the latency period (seconds) for arriving at the center of the open field. The box plots depict the mean (horizontal bar), 95% confidence limits (upper and lower boundaries of the box), and range (stems) corresponding to each parameter measured. Data were analyzed by one-way ANOVA with post hoc Tukey tests. Significant P-values and trends (italicized) are shown in the panels.

The OF test provides a measure of anxiety marked by prolonged latency in arriving at the center, less time spent in the center, and fewer entries into the center of the test area. Ethanol-fed rats spent lower mean percentages of time in the field center, and exhibited lower percentages of entries into the field’s center relative to controls. However, among ethanol-fed rats, myriocin treatment increased the mean percentage of time spent in the field’s center and the frequency of field center entries relative to vehicle, but did not fully normalize behavior (Figure 3). The CV and CM groups exhibited similar performance by OF testing. The mean latencies for arriving at the field centers overlapped and were not significantly different among the four groups due to large standard deviations.

### 3) Novel Object Recognition test (NOR):

**Figure 4.**
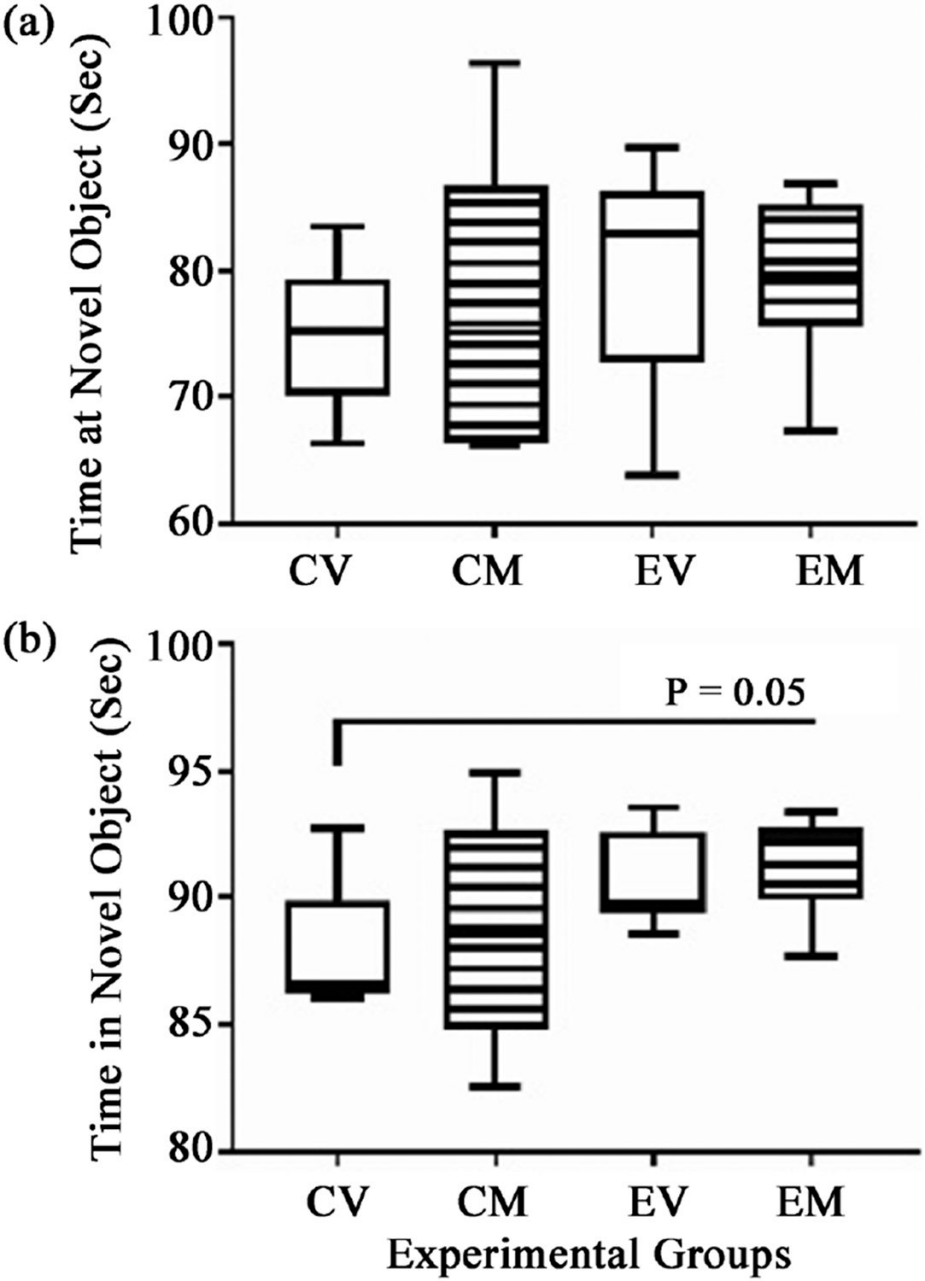
Novel Object Recognition (NOR) Testing: The 4-way, 8-week model generated in male and female Long Evans rats (see Figure 1) was sub-grouped as follows: CV = control diet + vehicle; CM = control diet + myriocin; EV = ethanol diet + vehicle; EM = ethanol diet + myriocin. NOR testing was assessed on Monday of Experimental Week 8 and data were electronically recorded and analyzed (Ethovision 13.0 software) with respect to the (a) time spent at the novel object and (b) time spent investigating (In) the novel object. The box plots depict the mean (horizontal bar), 95% confidence limits (upper and lower boundaries of the box), and range (stems) corresponding to each parameter measured. Data were analyzed by one-way ANOVA with post hoc Tukey tests. Significant P-values are indicated.

NOR testing was used to assess recognition learning and memory. Scoring was based on the number of times and percentage of time the rats spent investigating a novel versus familiar object. Although the mean duration and frequency of novel object investigation were lowest in the CV group, the differences from the other three groups were not statistically significant with respect to time spent at the novel object (Figure 4(a)), and with the exception of EM, the responses were similar in relation to the time spent investigating the novel object (Figure 4(b)). However, myriocin treatment of ethanol-exposed rats significantly increased the time investigating the novel object relative to CV controls (Figure 4(b)).

### 4) Morris Water Maze:

**Figure 5.**
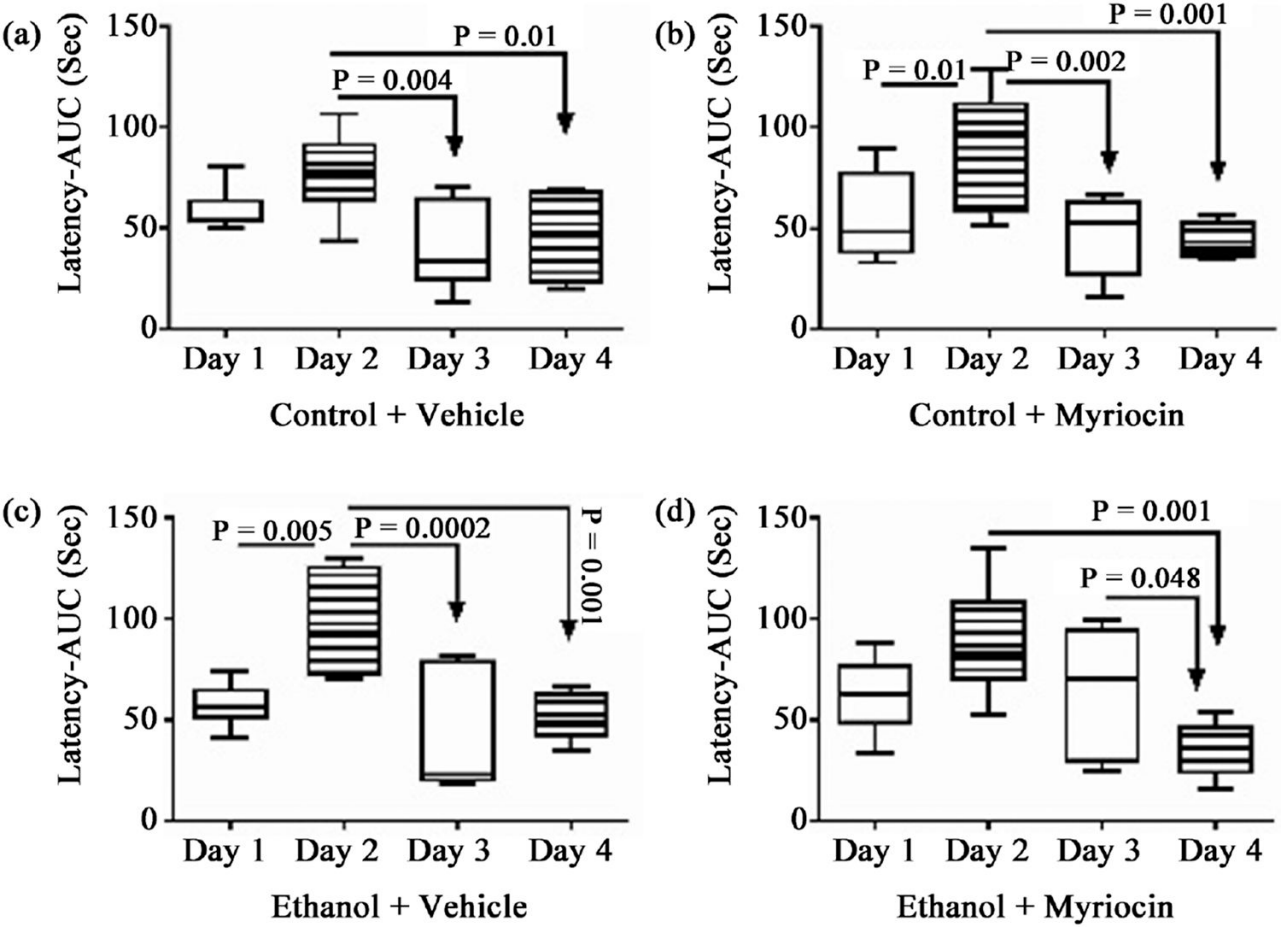
Morris Water Maze (MWM): The 4-way, 8-week model generated in male and female Long Evans rats (see Figure 1) was sub-grouped as follows: control diet + vehicle; control diet + myriocin; ethanol diet + vehicle; ethanol diet + myriocin. MWM testing was performed on four consecutive days (Tuesday through Friday) with three trials per day in the mornings of Experimental Week 8. Latencies to locate and land on the platform were electronically captured and analyzed (Ethovision 13.0 software). Box plots depict the mean area-under-the-curve (AUC) calculated performance over the 3 daily trials. Data were analyzed by one-way ANOVA with post hoc Tukey tests. Significant P-values are shown in the panels.

MWM testing of spatial learning and memory demonstrated similar trial-related trends in all groups such that the mean latencies to locate and land on the hidden platform were highest on Day 2, *i.e.* the acquisition phase of learning and memory, and declined sharply on Days 3 and 4 (Figure 5). Correspondingly, the mean latencies were significantly longer on Day 2 compared with Days 3 and 4 within each experimental group. Inter-group comparisons revealed significantly longer mean latencies in the EV relative to CV and CM, but not between EM and CV, CM, or EV on Trial Day 2 (Figure 2). On Days 3 and 4, CV, CM and EV had similar mean latencies whereas for EM, performance significantly improved with further reduction in mean latency between Trial Days 3 and 4 (Figure 2**(d)**). In addition, for EM, the mean latency on Trial Day 4 was comparable to those measured in CV and CM, and lower than in EV.

### 5) Ethanol Effects on White Matter Sphingolipids:

**Figure 6.**
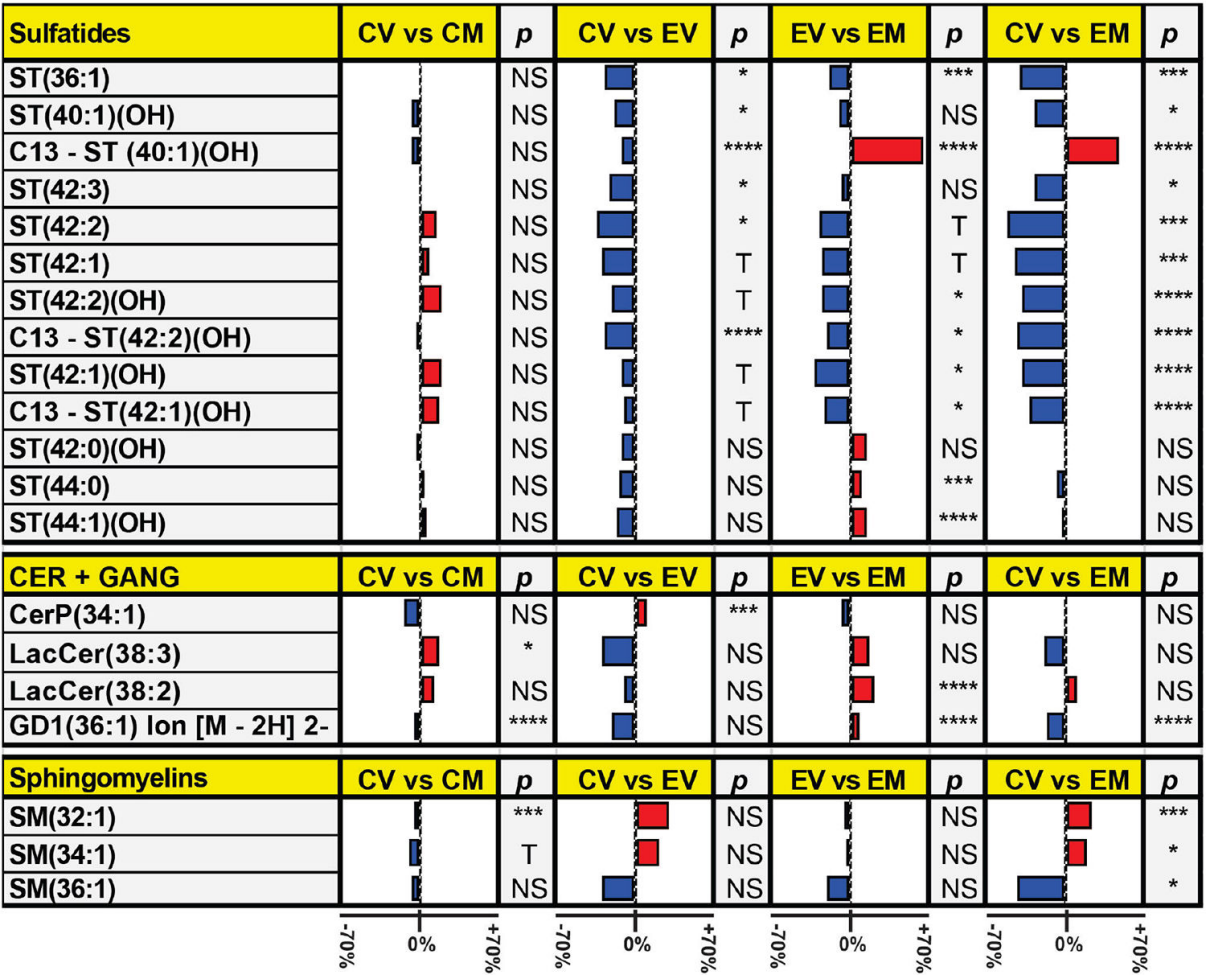
Ethanol and Myriocin effects on Frontal White Matter Sphingolipids. MALDI (negative ion mode) was used to detect and quantify lipid expression in frontal lobe white matter of control diet + vehicle (CV); control diet + myriocin (CM); ethanol diet + vehicle (EV); ethanol diet + myriocin (EM) treated Long Evans rats. Databar plots illustrate the calculated percentage differences in mean sphingolipid expression. Red bars show increased while blue bars show reduced lipid expression. Data analysis was restricted to sphingolipids expressed in all groups and included sulfatide (ST), C13-isotopes of ST, ceramide (Cer) including phosphorylated (CerP) and Lactosylceramide (Cer), ganglioside (GD1), and sphingomyelin (SM). Inter-group comparisons of mean lipid expression were made by T-test with 1% false discovery rate correction. P-values reflect differences in mean lipid expression indicated as: NS = not significant; T = trend (0.05 < P < 0.10); *P < 0.05; **P < 0.01; ***P < 0.001; ****P < 0.0001.

Results were aligned and compared based on detectable expression of the same lipids in all samples. Lipids not detected in all samples were excluded from the analysis. We detected 10 sulfatides (ST) plus 3 C13 isotopes of sulfatide, 1 phosphoceramide, 2 lactosylceramides (LacCer), 1 ganglioside (GD1), 3 sphingomyelin species (SM). Comparisons reflecting the mean percentage inter-group differences in the levels of lipid expression between CV and EV, CV and CM, EV and EM, and CV and EM are depicted in databar plots (Figure 6). Bars to the left of the vertical axis indicate relative reductions in lipid expression and bars to the right show increases in lipid expression. The mean expression levels of all 13 ST (including the 3 C13 isotope forms), 3 of the 4 ceramides/gangliosides, and 1 of 3 SMs were reduced by EV exposures, whereas 1 Cer and 2 SM lipids were higher in EV than CV. In addition, both LacCers, the single GD1, and 1 of the 3 SMs (36:1) were also reduced by EV whereas 2 SMs and 1 PC were increased by ethanol.

### 6) White Matter Responses to Myriocin:

**Figure 7.**
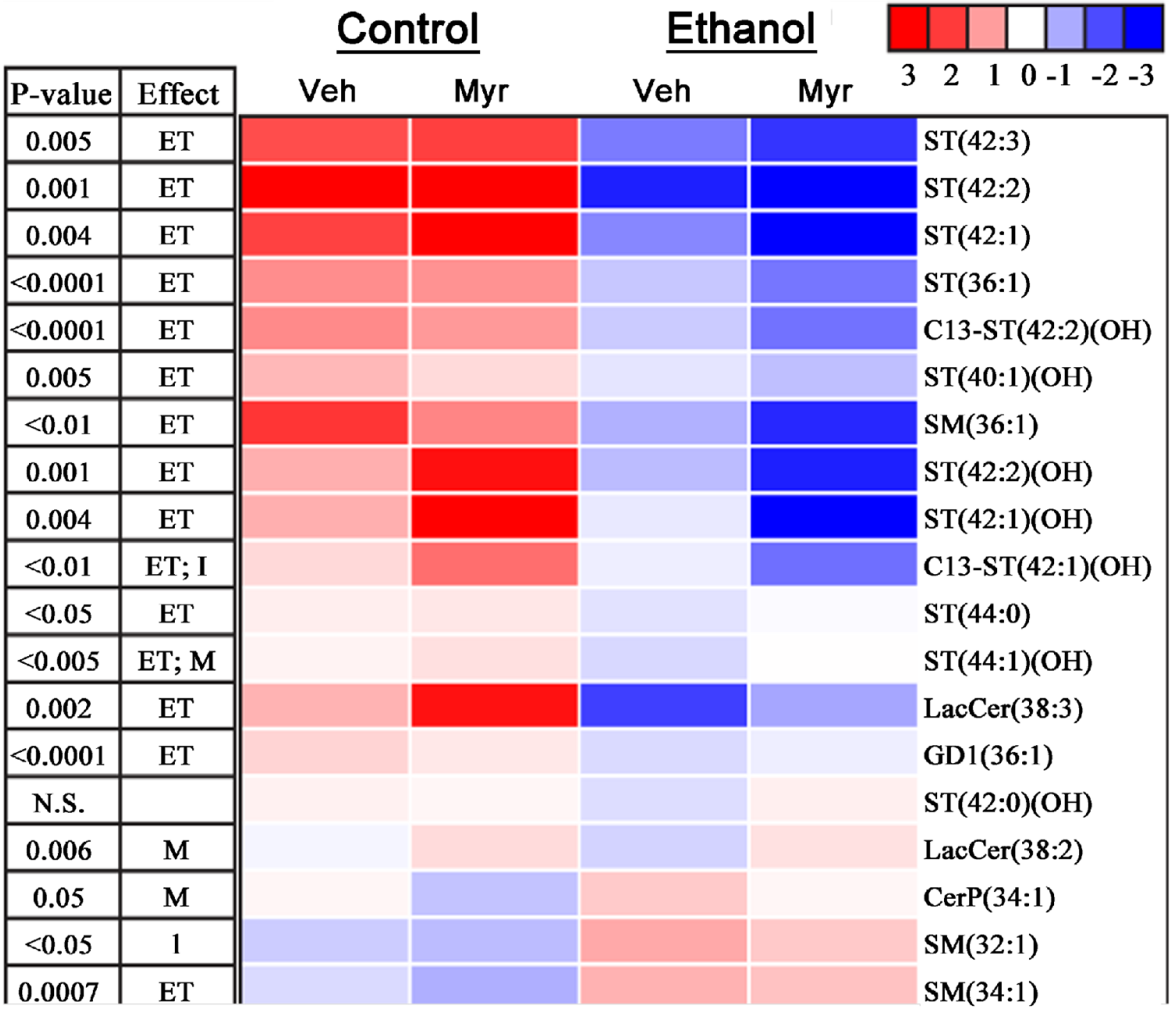
Heatmap display of myriocin (Myr) and ethanol effects on frontal lobe white matter sphingolipid expression as revealed by MALDI (negative ion mode). A 4-way Long Evans rat model of chronic + binge ethanol or control diet feeding plus myriocin or vehicle treatment was used in these studies. The mean levels of sulfatide (ST), C13-isotopes of ST, ceramide (Cer) including phosphorylated (CerP) and Lactosylceramide (Cer), ganglioside (GD1), and sphingomyelin (SM) that were detected in all groups were used to generate the heatmap in Treeview. Ion intensities are displayed using a 7-color palette corresponding to z-scores scaled to have a mean of 0 and standard deviation of 3.0. m/z values appear on the right-hand side. Inter-group comparisons were made using two-way ANOVA tests. Corresponding P-values are indicated. In addition, the factor responsible for the significant difference (effect) is listed (ET = ethanol, M = Myriocin; I = interaction between ethanol and myriocin).

Principal component analysis (PCA) of sphingolipid expression profiles in frontal lobe white matter revealed tighter clustering of CV with CM, and EV with EM than either control with either ethanol-exposed group, indicating a dominant overall effect of ethanol compared with myriocin (Supplementary Figure S1). Nonetheless, distinct additive effects of myriocin were detected by heatmap displays and Two-way ANOVA tests (Figure 7). Myriocin treatment of control rats increased frontal white matter expression of 4 ST and one C13 isotope of ST and both LacCers. Otherwise, the effects were modest. The main effects of myriocin in ethanol-fed relative to EV and CV were to increase expression of C13 ST(40:1)(OH). In addition, myriocin increased expression of ST(42:0)(OH), ST(44:0) and ST(44:1)(OH), LacCer(38:3) and LacCer(38:2) relative to EV, similar to the effects of CM compared with CV. Comparisons between CV and EM largely mirrored the effects of EM versus EV with regard to ST, but relative effects on SM and ceramide expression were modest (Figure 7).

## 4. Discussion

### 1) Study Goals and Design:

This study examined the therapeutic effects of myriocin, a potent ceramide inhibitor, on alcohol-related biochemical neuropathology, focusing on its capacity to improve the integrity of white matter myelin and associated neurobehavioral functions. Long Evans rats were used in a well-established 8-week, 4-way model of chronic + binge ethanol in which the animals were fed liquid diets containing 0 or 24% ethanol for 8 weeks and were binged with ethanol or saline for the last 3 weeks; a subgroup of the control and ethanol groups were treated with myriocin for the last 6 weeks. The chronic + binge model produces cerebral white matter degeneration [11], mimicking the effects of heavy drinking in humans [1] [5].

### 2) Gender Effects:

Earlier studies showed that chronic ethanol exposures lead to a disproportionate loss of cerebral white matter with impairments in neurocognitive function. As expected, the ethanol-fed groups had lower mean brain weights upon sacrifice; however, the variance was higher in EM compared with EV. The subgroup analysis revealing gender-related effects such that EV females had the smallest, *i.e.* most atrophic brains is not readily explained, but is consistent with prior evidence that females are more vulnerable than males to the neurotoxic and neurodegenerative effects of heavy alcohol consumption [52] [53]. The findings herein also demonstrate the preventive or rescue effects of myriocin on brain structure. Correspondingly, ethanol exposures caused deficits in learning and memory, but myriocin partially normalized performance on the NOR and MWM tests.

### 3) Therapeutic Effects of Myriocin:

NOR and MWM assess recognition learning and spatial learning and memory respectively; typically, performance in these tests has been correlated with hippocampal function [20] [54]. However, growing evidence suggests that impairments in these modalities are likely mediated by metabolic dysfunction linked to neurodegeneration [55] [56] [57]. In a previous study of chronic alcohol-mediated liver disease, myriocin remediated hepatic structural and metabolic anomalies via disruption of ceramide synthesis mechanisms [26]. Although there is no definite evidence that myriocin crosses the blood-brain barrier (BBB), its small size and partial lipophilic structure make direct access to the central nervous system highly likely [58]. Within our model of alcohol-related white matter degeneration, the myriocin-associated improvements in neurobehavioral test performance corresponded with the increased brain weights in ethanol-exposed rats, supporting the concept that myriocin prevents degeneration or restores the structural integrity of white matter following heavy ethanol exposure.

### 4) Adverse Effects of Chronic + Binge Alcohol on White Matter Sphingolipids:

White matter is largely composed of lipid-rich myelin and is especially vulnerable to alcohol-related injury and degeneration. Sphingolipids, including sulfatides (which comprise 4% of total myelin lipids), sphingomyelin and glycosphingolipids, together with cholesterol, are major components of white matter myelin [59]. A study published in 2005 showed that ethanol exposures significantly alter white matter lipid profiles such that ceramide content is increased [60]. Subsequently, heavy alcohol exposures were found to reduce matter myelin sulfatides and sphingomyelin and increase ceramides [61]. Although low physiologic levels of ceramides mediate normal brain metabolism and function, elevated toxic accumulations can cause demyelination and cell death [19] [20]. Moreover, the potential consequences of sulfatide depletion were highlighted by its association with ethanol-mediated neurodegeneration in humans [10] and experimental models [11], and the earliest stages of Alzheimer’s neurodegeneration [62].

### 5) Therapeutic Efficacy of Myriocin as a Reversal Agent for Alcohol-Medicated White Matter Biochemical (Sphingolipid) Pathology.

Myriocin, a serine palmitoyltransferase inhibitor, reduces ceramide buildup [26], and therefore may provide remediation of diseases linked to matter myelin degeneration associated with altered sphingolipid profiles. A major objective of this research was to use MALDI-IMS to determine if myriocin treatment could ameliorate or reverse alcohol-mediated white matter sphingolipid biochemical pathology vis-à-vis continued chronic + binge exposures. The results demonstrated that ethanol reduced sulfatide and sphingomyelin expression and that myriocin partially abrogated these effects by normalizing matter myelin levels of ST(44:0), ST(44:1)(OH), and ST(42:0)(OH), although opposing effects occurred with respect to SM(32:1) and SM(34:1). In addition, alcohol-associated increases brain ceramide-1-phosphate levels, which also correlate with neurodegeneration [63], were reduced by myriocin, as illustrated by the lowered level of CerP(34:1) (see heatmap in Figure 7). Together, the findings suggest that the adverse effects of heavy alcohol consumption on matter myelin sphingolipid composition and associated neurobehavioral pathology can be ameliorated at least in part by myriocin treatment.

### 6) Potential Alternative Therapeutic Strategies:

Of note is that abstinence can partially remediate the adverse effects of alcohol on cerebral white matter atrophy with altered sphingolipid expression, but the responses are incomplete [11]. However, in general, it is difficult to permanently halt heavy drinking behavior, particularly since heavy drinking may exacerbate behaviors that drive alcohol use disorders to progress [64]. Without effective intervention, chronic alcohol-induced neurodegeneration progresses. However, the extent to which the atrophy remains at least partially reversible after a prolonged period of heavy drinking is unknown.

### 7) Forward View of Therapeutics for Alcohol-Related Brain Degeneration:

A key aspect of these results is that myriocin’s therapeutic effects occurred despite continued heavy ethanol exposures, which has relevance to humans with poorly controlled alcohol use disorders. Furthermore, the findings suggest that myriocin-associated neurobehavioral improvements are associated with structural and metabolic repair in cerebral white matter. By extension, therapeutic amelioration of alcohol-mediated brain injury may enhance performance in other modalities such as those pertaining to social functions. Future studies will directly assess the degrees to which myriocin restores the structural integrity of white matter myelinated fibers following chronic heavy ethanol exposures, and the maximum severity injury that can be sustained and still be remediable by myriocin treatment.

## Supplementary Material

1
