# Supplementary material for: Therapeutic Effects of Myriocin in Experimental Alcohol-Related Neurobehavioral Dysfunction and Frontal Lobe White Matter Biochemical Pathology
Source: J Behav Brain Sci. Author manuscript; Available in PMC 2023 Feb 21. (PMC9942847; doi:10.4236/jbbs.2022.122003)
Supplement: 1 [file NIHMS1869477-supplement-1.pdf]

Supplemenary

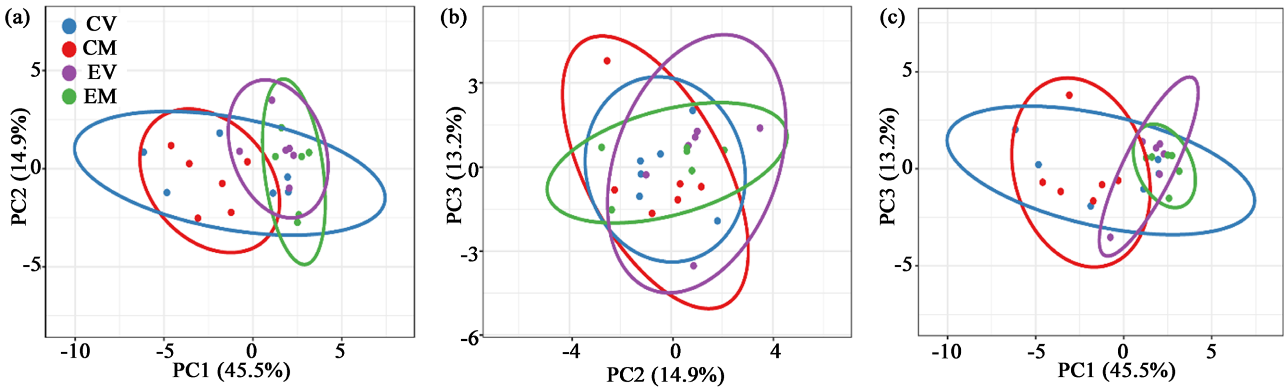

Figure S1. Principal Component Analysis (PCA) Plots. PCA plots of sphingolipids detected in frontal white matter by MALDI-TOF in the negative ionization mode. Sphingolipids detected between 600 and 2000 Da mass range were compared among the sub-groups included in the 4-way Long Evans rat model: control diet + vehicle (CV); control diet + myriocin (CM); ethanol diet + vehicle (EV); ethanol diet + myriocin (EM). Two dimensional PCA plots displaying (a) PC1 × PC2, (b) PC2 × PC3, and (c) PC1 × PC3 were generated in ClustVis software. X and Y axes show PC1, PC2, and PC3 that correspond to 45.5%, 14.5%, and 13.2% of total variance, respectively. PC1 and PC3 show dominant clustering of CV with CM and EV with EM, corresponding with the primarily ethanol effect observed by two-way ANOVA (Figure 7).
